# Sphingosine-1-phosphate signaling in *Leishmania donovani* infection in macrophages

**DOI:** 10.1371/journal.pntd.0006647

**Published:** 2018-08-17

**Authors:** Mohd Arish, Atahar Husein, Rahat Ali, Shams Tabrez, Farha Naz, Mohammad Zulfazal Ahmad, Abdur Rub

**Affiliations:** 1 Infection and Immunity Lab (414), Department of Biotechnology, Jamia Millia Islamia (A Central University), New Delhi, India; 2 Centre for Interdisciplinary Research in Basic Science, Jamia Millia Islamia (A Central University), New Delhi, India; 3 Department of Medical Laboratory Science, College of Applied Medical Sciences, Majmaah University, Al Majmaah, Saudi Arabia; McGill university, CANADA

## Abstract

**Background:**

Sphingosine-1-phosphate (S1P) is a crucial regulator of a wide array of cellular processes, such as apoptosis, cell proliferation, migration, and differentiation, but its role in *Leishmania donovani* infection is unknown.

**Methodology/ principal findings:**

In the present study, we observed that *L*. *donovani* infection in THP-1 derived macrophages (TDM) leads to decrease in the expression of *S1pr2* and *S1pr3* at mRNA level. We further observed that *Leishmania* infection inhibits the phosphorylation of sphingosine kinase 1 (sphK1) in a time-dependent manner. Exogenous S1P supplementation decreases *L*. *donovani* induced ERK1/2 phosphorylation and increases p38 phosphorylation in TDM, resulting in a decrease in the intracellular parasite burden in a dose-dependent manner. On the other hand, sphK inhibition by DMS increases ERK1/2 phosphorylation leading to increased IL-10 and parasite load. To gain further insight, cytokines expression were checked in S1P supplemented TDM and we observed increase in IL-12, while decrease IL-10 expression at mRNA and protein levels. In addition, treatment of antagonist of S1PR2 and S1PR3 such as JTE-013 and CAY10444 respectively enhanced *Leishmania*-induced ERK1/2 phosphorylation and parasite load.

**Conclusions:**

Our overall study not only reports the significant role of S1P signaling during *L*. *donovani* infection but also provides a novel platform for the development of new drugs against Leishmaniasis.

## Author summary

*Leishmania donovani* is an intracellular parasite which is internalized by host macrophages by subverting several intracellular signaling events. During infection suppression of p38 MAPK and activation of ERK1/2 MAPK have been acclaimed for survival and proliferation of these protozoan parasites. In this study, we show novel signaling pathways that interact with these MAPK that further contributes to determine the final fate of the disease. Sphingosine-1-Phosphate (S1P) is a bioactive lipid that binds to a family of G-protein coupled receptors known as S1P receptors. TDM infected with *Leishmania donovani* showed a decrease in the expression of S1PR1-3. Moreover, the enzyme that catalyzes S1P production, Sphingosine Kinase 1, showed decreased activation in a time-dependent fashion in infected cells. Furthermore, exogenously supplementation of S1P clears intracellular parasite burden by a decrease in ERK1/2 phosphorylation and IL-10 at mRNA and protein level. On the other hand, S1P induces anti-leishmanial response by activating p38 phosphorylation and IL-12 at mRNA as well as at protein level. To further gain insight on the receptors subtypes involved in the anti-leishmanial response, we specifically blocked S1PR2 and S1PR3. In this study, we found a tremendous increase in the parasite load as a result of increased IL-10 secretion and ERK1/2 phosphorylation on combination of these inhibitor doses. Taking together, our study thus suggested the possible involvement of S1PR2-3 during *Leishmania donovani* infection in human macrophages. These findings thus elaborate our knowledge in understanding the interaction of signaling intermediates during *Leishmania* infection which may lead to the discovery of novel therapeutic interventions.

## Introduction

Leishmaniasis is a neglected tropical disease that affects about 12 million people worldwide [1] It is caused by intracellular protozoa parasite *Leishmania* that invades macrophages and selectively impairs host’s critical signaling pathways for its successful intracellular growth and proliferation [2]. In particular, alteration in lipid metabolic pathways, lipid relocation, modification, and accumulation during *Leishmania* infection has been proven to be a critical step in the progression of disease [3]. In addition, *Leishmania* infection leads to increase in ceramide generation that further depletes cholesterol from the membrane and disrupts lipid rafts resulting in weak CD40 mediated signaling that leads to increased ERK1/2 phosphorylation and impaired antigen presentation to the T cells, worsening the diseased condition [4,5]. S1P signaling is emerging as a prominent regulatory pathway in cells that governs myriad of downstream signaling events [6]. The cascade begins with the generation of S1P from sphingosine by the action of sphK. Afterward, S1P translocates to the outer membrane and binds to its receptors, namely S1PR1-5, which triggers the small G-proteins associated with them [7]. These G-proteins then activates different signaling proteins resulting in numerous effectors functions. Being such a crucial regulator of cellular processes, it has been seen that this signaling pathway often dysregulated during several diseases [8,9]. In addition, the possible therapeutic strategy can be designed by carefully monitoring the impairment of this signaling during several diseased conditions [8,9].

S1P signaling has been well established in bacterial and viral diseases. Till date, no study addresses the role of S1P signaling in *Leishmania donovani* infection in human macrophages, The role of S1P in another intracellular pathogen such as *Mycobacterium* has been well studied *in vitro* and *in vivo* [10]. It was documented that S1P possess antimycobacterial properties such as reduction in intracellular growth by enhancement of phagolysosome acidification, induction of IFN-γ and enhanced antigen processing and presentation in monocytes [10,11]. Apart from this, it was shown that host sphingosine kinase regulates antimycobacterial responses and its inhibition leads to sensitization of RAW 264.7 macrophage to infection due to reduced expression of anti-mycobacterial effector functions such as pp38, inducible nitric oxide synthase (iNOS) and Lysosome-associated membrane protein 2 (LAMP 2) [12]. In *Bordetella pertussis* infection in mice, S1P mediated signaling through S1PR resulted in reduced pathology due to the infection [13]. Similarly, during *Yersinia pestis* infection, activation of S1PR1 mediated signaling by SEW2871 limits intra-nodal trafficking of infection [14]. Alveolar macrophages from sphK1-knockout mice showed an increased burden of intracellular fungal, *Cryptococcus neoformans* [15], which suggests a protective role of S1P against *C*. *neoformans* infection. Currently, the role of S1P is unknown during *Leishmania donovani* infection in human macrophages. Also, the involvement of S1PR mediated signaling during *Leishmania* infection is not studied till date. Hence, in our study, we examined the role of S1P signaling during *Leishmania donovani* infection that should be helpful for generation of host-directed therapies against Leishmaniasis.

To explore the role of S1P signaling in *Leishmania donovani* infection we first analyzed the expression of S1PR1-5 in infected and uninfected TDM. We next checked the phosphorylation of sphK1, the enzyme responsible for S1P production, in both TDM and human monocyte derived macrophages (hMDM). For further studies, we checked the phosphorylation of MAPK such as ERK1/2 and p38 in presence of S1P in infected and uninfected macrophages. In addition, cytokines such as Interleukin (IL) 10 and IL-12 were checked at mRNA and protein levels and parasite load was checked in infected macrophages upon S1P supplementation. For further confirmation of our results ERK1/2 and p38 activation was also studied upon inhibition of sphK or S1P supplementation in hMDM As observed earlier, the expression of S1PR2 and S1PR3 were decreased during the infection, we checked for ERK1/2 phosphorylation, cytokine secretion and parasite load in the presence of S1PR2 and S1PR3 inhibitors, JTE-013 and CAY10444, respectively.

## Materials and methods

### Ethical statement

The study was approved by Institutional Ethical Committee (IEC), Jamia Millia Islamia, New Delhi for the human subject participation. Each donor provided written informed consent for the collection of blood and subsequent analysis.

### Reagents

RPMI 1640, M199, Fetal Bovine Serum (FBS), and penicillin and streptomycin were purchased from Life Technologies. DMS (N-N Dimethyl-sphingosine), JTE-013 and CAY10444 were purchased from Cayman chemicals. S1P was purchased from Tocris. Antibodies such as ERK1/2, phospho-ERK1/2, p38, and phospho-p38 were purchased from Cell Signaling Technology. Phospho-sphingosine kinase 1 and total sphingosine kinase 1 antibody was purchased from ECM biosciences. ELISA kit for IL-0 and IL-12 were purchased from BD Biosciences.

### Parasite

The standard strain of *L*. *donovani*: (MHOM/IN/83/AG83) was maintained in M199 media (Life Technologies) with 25mM HEPES (Sigma) and supplemented with 10% heat-inactivated Fetal bovine serum (Life Technologies) with 1% penicillin and streptomycin (Life Technologies) at 22°C. Fourth to fifth-day culture was used to infect differentiated TDM or hMDM.

### Human macrophage cell line

The THP-1 cell line was maintained in RPMI 1640 medium (Life Technologies) supplemented with 10% heat-inactivated FBS (Life Technologies) and 1% streptomycin-penicillin (Life Technologies) at 37°C in 5% CO_2_. PMA (phorbol 12-myristate 13-acetate; Sigma) was used for differentiation of THP-1 cells into macrophages by incubating cells for 24 hrs with 5 ng/ml PMA at 37°C in 5% CO_2_ in flat-bottom 6-well tissue culture plates (BD Biosciences).

### Peripheral blood mononuclear cells isolation

Peripheral blood mononuclear cells (PBMCs) were isolated from buffy coats obtained from healthy donors (M.A Ansari Health Center, Jamia Millia Islamia, New Delhi, India) on Histopaque (Sigma). PBMCs were seeded on 6 wells plate and allowed to adhere. For differentiation of hMDM, non-adherent cells were removed by gentle washing and adherent cells were incubated with 5ng/ml GM-CSF (Pepro-Tech) at 37°C in 5% CO_2_ for 24 hrs and replenished with supplemented RPMI 1640 containing 10% FBS for 6 to 7 days.

### Infection of macrophages

Macrophages were harvested and distributed into six-well plates at 2 × 10^6^ cells/well. Stationary-phase promastigotes were added to differentiated cells at an infection ratio of 1:10 to for 6 hrs initiate infection. Infected macrophages were further replenished with supplemented RPMI 1640 containing 10% FBS for additional 42 hrs for different studies.

### MTT assay

1×10^5^ cells per well were seeded onto 96-wells plate and was treated with PMA for diiferentiation for 24 h. Next day cells were washed and media was replaced with fresh media and cultured for additional 24 h for resting. After 24 h the cells were treated with different inhibitor used in the study DMS (5 μM), JTE-013 (10 μM), and CAY10444 (10 μM) for 42 hours. Cell viability was determined using the MTT cell viability assay. 3-(4,5-Dimethyl-2-thiazolyl)-2,5-diphenyl-2H-tetrazolium bromide, MTT (Sigma–Aldrich) was applied at in dark following 4 h incubation at 37°C. The MTT containing medium was replaced with 100 μl of isopropanol-HCl (0.1N) and kept at 37°C for 10 min to solubilize the formazan crystals. The samples were transferred to 96-well plates and the absorbance of the converted dye was measured at 570nm. The percent cell viability of the control (non-treated) cells was taken as 100%.

### RT-PCR and real-time PCR

RNA was extracted by Trizol (Sigma) as per user’s information and was quantified on Biophotometer (Eppendorf, Germany) and 1 μg of RNA was used to prepare cDNA. Levels of *Il-10* and *Il-12* expressions were determined in the treated and untreated IM by quantitative PCR (qPCR), with β-Actin taken as an endogenous control (Primer sequences in S1 Table). q-PCR was carried out in a final volume of 10μL in Lightcycler 480 (Roche). The reactions were carried out with an initial denaturation step of 10 minutes at 95°C, followed by 40 cycles of denaturation, for 15 seconds, at 95°C, and annealing/extension for 1 minute, at 60°C. Relative gene expression was analyzed by the Livak method.

### Parasite load

For parasite load, TDM were seeded on sterile coverslips placed in 6 well culture plates. Infection was given at 1:10 for 6 hrs and infected cells were treated with different concentration of inhibitors/ S1P and cultured for next 42 hrs. Infected cells were washed with PBS and then fixed with ice-cold methanol for 10 min and air dried. Wells were immersed in Giemsa stain for 45 min and washed 2–3 times with PBS. At least 200 cells were observed from a minimum of 15 randomly selected fields for each condition to determine the average number of parasites per macrophage. The parasite load was calculated in percent for the different condition after taking parasite load in control as 100%.

### Protein extraction and western blotting

After treatment, macrophages were washed twice with PBS and lysed with ice-cold lysis buffer (50 mM Tris-HCl, [pH 7.4], 150 mM NaCl, 1% Triton-X, 1 mM Sodium Orthovanadate, 10 mM Sodium Fluoride, 1X protease inhibitor cocktail (Cell Signaling Technology). Lysates were centrifuged at 14,000 × g at 4°C for 15 min, and the resulting supernatants were transferred to fresh tubes and stored at −80°C until required. 40–50 μg of protein were used for western blotting.

### Enzyme-linked immunosorbent assay

IL-10 and IL-12-specific enzyme-linked immunosorbent assay (ELISA) was performed to detect the level of secreted IL-10 and IL-12 in the cell-free supernatant obtained from different experiments using ELISA kits as per manufacturer’s instructions.

### Densitometric analysis

Immunoblots and PCR products were analyzed using ImageJ software, National Institute of Health, version 1.50i. The band intensity was calculated and normalized to corresponding control.

### Statistical analysis

The results shown are representation from a minimum of three similar experiments which generated reproducible data. The statistical analysis was performed using GraphPad Prism, version 6.0 (GraphPad, San Diego, CA, USA). *P*-value of less than 0.05 was considered significant. The error bars of the values represent ± SD from the replicates. Tukey's multiple comparisons test and Student t-test were performed to ascertain the significance of the differences between the means of the control and the experimental groups.

## Results

### Expression of *S1pr1-5* in infected and uninfected human macrophages

**Fig 1:**
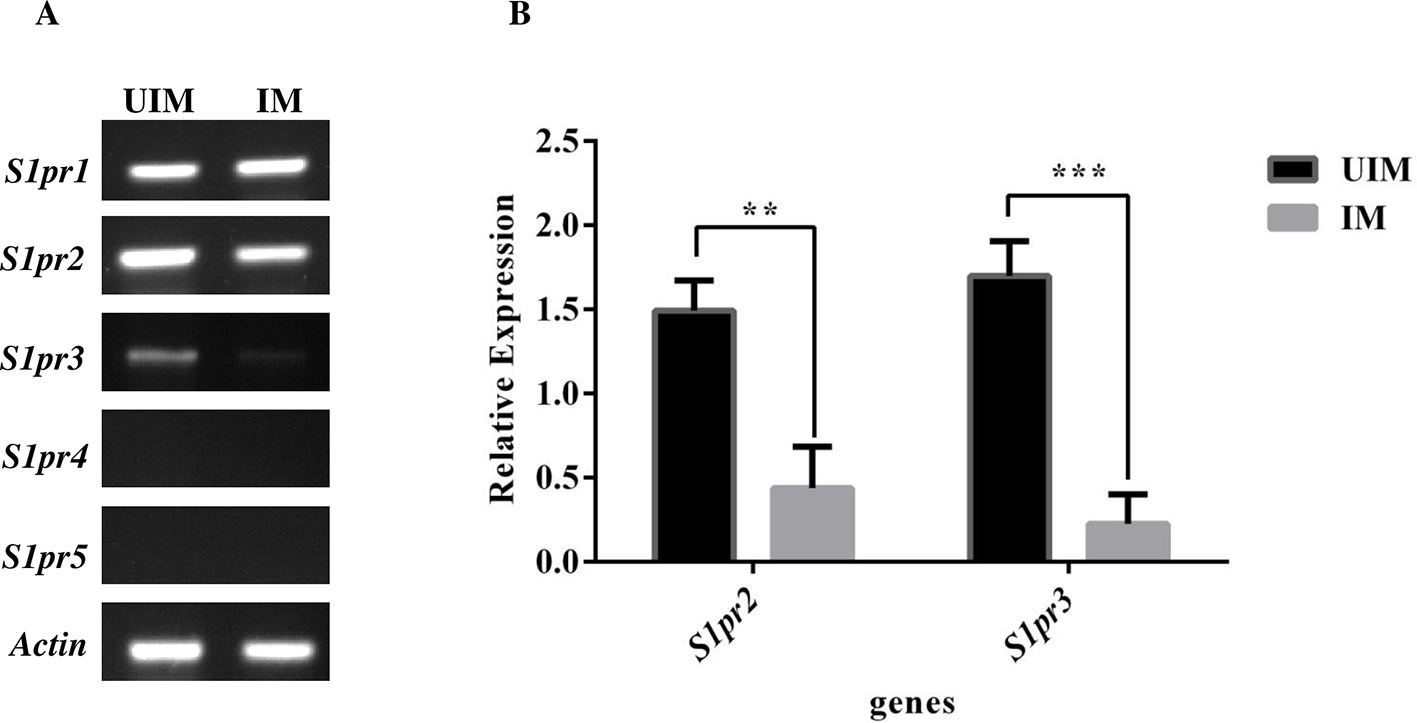
Expression of S1PRs in *Leishmania donovani* uninfected macrophages (UIM) and infected macrophages (IM). TDM were cultured in six-well plates in the presence or absence of *L*. *donovani* infection (MOI  =  1:10) for 6 h, TDM were washed to remove non-internalized parasites and incubated for next 42 h. **A.** mRNA expression of S1PR1-5 and beta-actin was performed by semi-quantitative PCR. **B.** Relative expression of S1PR2 and 3 in UIM and IM after normalization with housekeeping gene beta-actin. The data is a representation of mean ± SD from three independent experiments**, p < 0.01 ***, p <0.001.

The expressions of *S1pr1-5* were checked upon *Leishmania donovani* infection in PMA differentiated human macrophages at mRNA level by semi-quantitative PCR. The macrophages were infection with *Leishmania donovani* promastigotes (multiplicity of infection [MOI] 1:10, macrophage to parasite) for 6 hours. The non-internalized parasite was removed by gentle washing 2–3 times with PBS. The macrophages were further for additional 42 hours. After the given time, mRNA was extracted and cDNA was prepared using 1μg RNA. In addition, we evaluated the fold change in the expression of these receptors using beta-actin as endogenous controls to normalize the S1P receptors gene expressions. We found the expression of *S1pr1-3* in both infected and uninfected macrophages, however, *S1pr*3 was detected at very low level (Fig 1A). In our study, we found that there was a significant decrease in the expression of *S1pr2* and *S1pr3* in infected macrophages (Fig 1B), while there was a not significant change in the expression pattern of *S1pr1* in infected macrophages. In addition, we couldn’t detect the expression of *S1pr4* and *S1pr5* in both infected and uninfected TDM (Fig 1A).

### Decrease in the sphingosine kinase 1 phosphorylation in infected human macrophages

**Fig 2:**
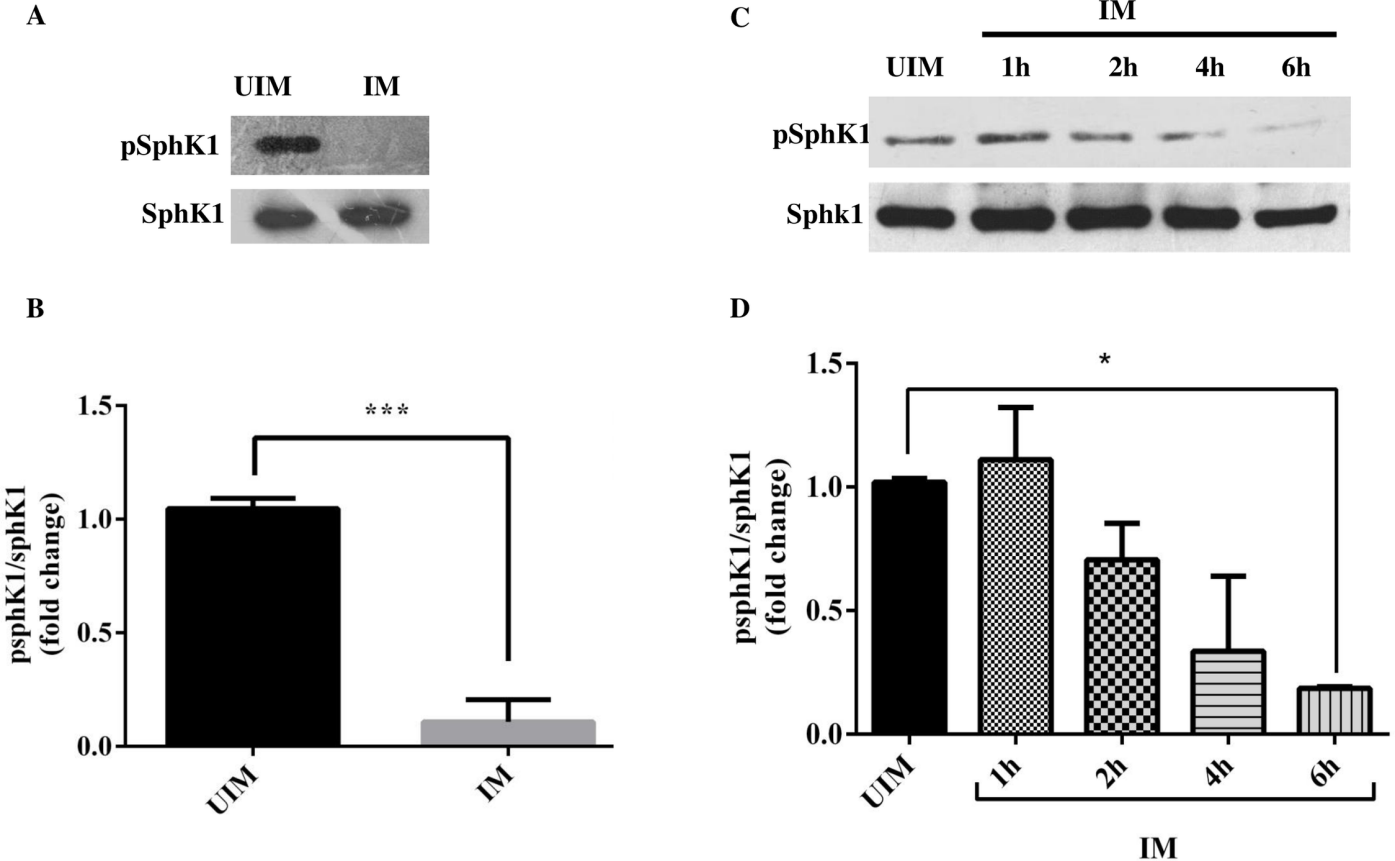
Sphingosine kinase 1 phosphorylation in UIM and IM. TDM were cultured in six-well plates in the presence or absence of *L*. *donovani* infection (MOI  = 1: 10) for 6 h, TDM were washed to remove non-internalized parasites and incubated for next 42 h. **A.** Western blot showing phosphorylation of sphK1 and total sphK1 in UIM and IM. **B**. Fold change in the phosphorylation of sphK1 during *L*. *donovani* infection at 48 hrs after normalization with total sphK1 in UIM and IM. **C.** Western Blot showing phosphorylation of sphK1 and total sphK1. **D.** Fold change in the phosphorylation of sphK1 during *L*. *donovani* infection at 1, 2, 4, and 6 hrs after normalization with total sphK1. The data is a representation of mean ± SD from three independent experiments *, p< 0.05;***, p < 0.001.

The level of sphK1 phosphorylation was checked in infected and uninfected TDM. The proteins were extracted from both the given experiment and analyzed by western blot for the detection of sphK1 phosphorylation. Total sphK1 was used as loading control for both the experiment. In our experiment, we observed that there was a significant decrease in the phosphorylation of sphK1 in the infected TDM (Fig 2A and 2B). Similarly, we also checked in the sphingosine kinase phosphorylation at various time intervals and we found a there was a time-dependent decrease in the phosphorylation level of sphK1 (Fig 2C and 2D). The decrease in the phosphorylation of sphK1 was also observed in hMDM 48h post infection (S1A and S1B Fig).

### S1P supplementation induces pro-inflammatory response in human macrophages

**Fig 3:**
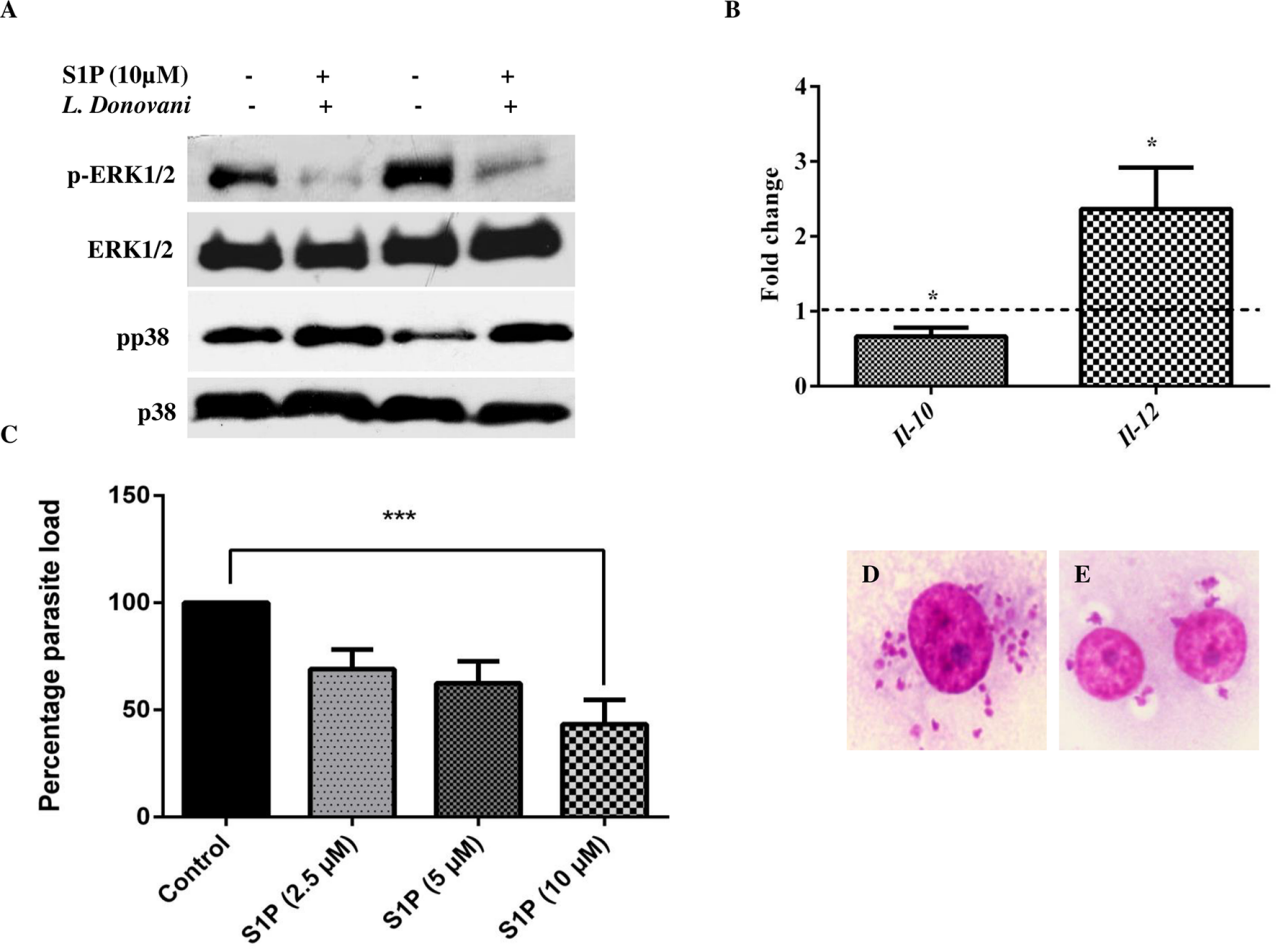
Anti-leishmanial response of S1P. TDM were cultured in six-well plates in the presence or absence of *L*. *donovani* infection (MOI  =  1:10) for 6 h, TDM were washed to remove non-internalized parasites and incubated for next 42 h in presence and absence of S1P**. A.** Western Blot showing phospho**-**ERK1/2, total ERK1/2, phospho-p38, and p38. **B.** The graphic indicates the fold change of mRNA levels of IL-10 and IL-12 in S1P treated infected macrophages in comparison to untreated infected macrophages by real-time PCR. Relative quantification was performed by the comparative Ct method (△△Ct). Giemsa stained *L*. *donovani* infected TDM. **C**. The % parasite load was determined by direct counting with an optical microscope. **D**. Control and **E**. S1P (10 μM) treated infected TDM. The data is a representation of mean ± SD from three independent experiments. *, p<. 0.05; ***, p < 0.001.

As sphK1 phosphorylation is necessary for S1P biosynthesis, we checked the effect of S1P supplementation on parasite load, pro-inflammatory and anti-inflammatory response. Firstly, we examined ERK1/2 and p38 activation in uninfected macrophages and infected TDM with *L*. *donovani* on S1P supplementation (10 μM) for 48 h. After given time, proteins were extracted and analyzed for the detection of phospho-ERK1/2 and phospho-p38. In our study, we found that S1P supplementation showed diminished ERK1/2 and increased p38 phosphorylation, as measured by western blot analysis (Figs 3A and S2A and S2B).

Next, we checked for the expression of cytokines such as *Il-10* and *Il-12* at mRNA level by both semi-quantitative PCR and real-time PCR. We found that S1P supplementation decrease *Il*-*10* expression and increases *Il*-*12* expression in infected macrophages (Figs 3B and S2C). In case of infected hMDM, we found that there was an increase in *Il*-*12* expression upon S1P supplementation. However, we also registered a nonsignificant change in *Il*-*10* expression at mRNA level (S4C Fig). We also determine the parasite load in presence of increasing doses of S1P and we found a dose-dependent decrease in the parasite burden in infected macrophages (Fig 3C). The most significant decrease in the parasite load was observed at maximum concentration i.e 10 μM (Fig 3C–3E).

### Pro-leishmanial response of sphingosine kinase inhibition by DMS

**Fig 4:**
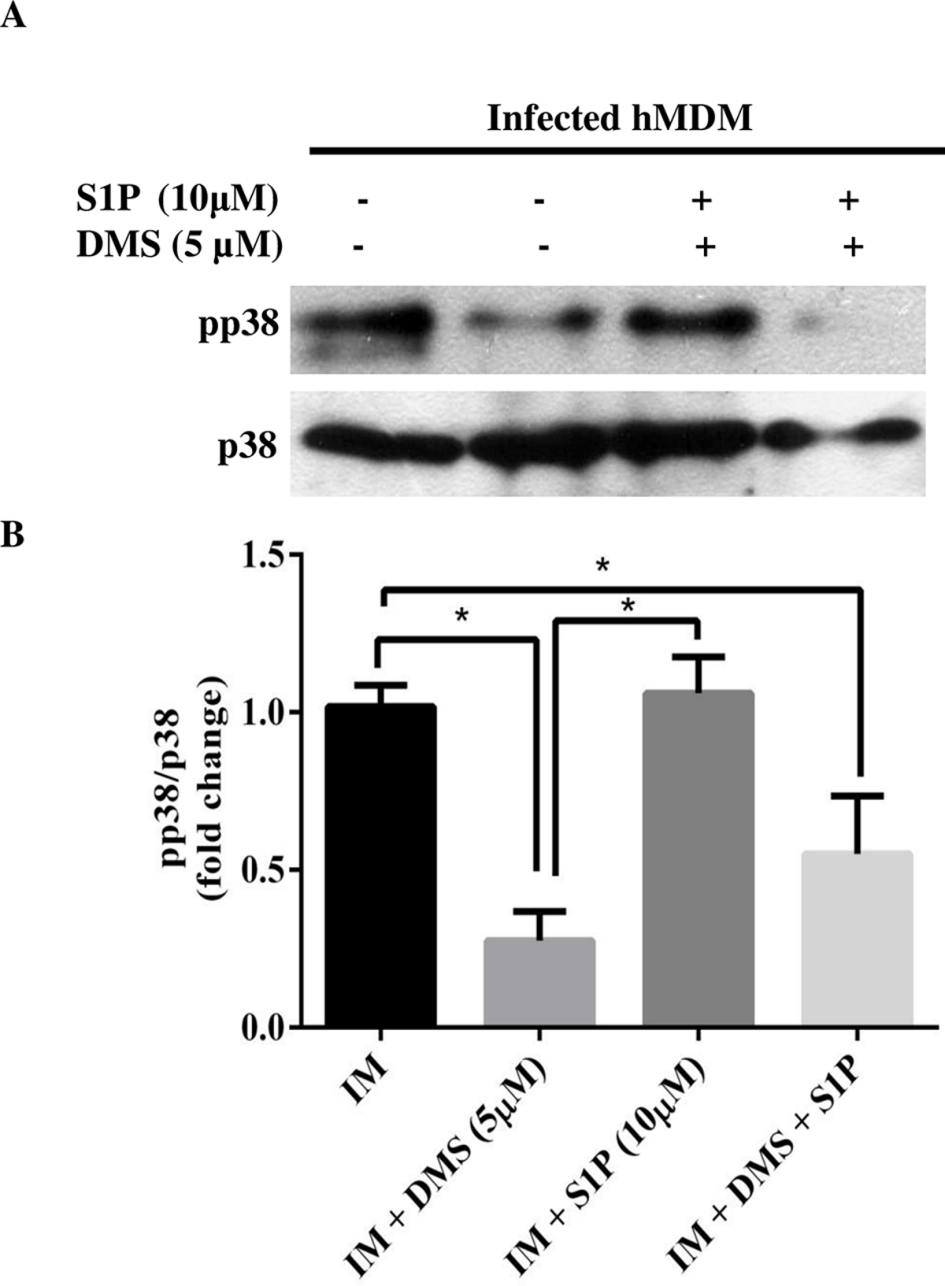
p38 phosphorylation in infected macrophages in presence of DMS, S1P or both in hMDM. hMDM were cultured in six-well plates in the presence or absence of *L*. *donovani* infection (MOI  =  1:10) for 6 h, hMDM were washed to remove non-internalized parasites and incubated for next 42 h in presence and absence of S1P, DMS or both. Western Blot showing **A.** phospho**-**p38 and total p38, **B.** Densitometric analysis of phospho-p38 in presence of DMS, S1P, or both in IM, after normalization with total p38. The data is a representation of mean ± SD from two independent experiments *, p< 0.05.

**Fig 5:**
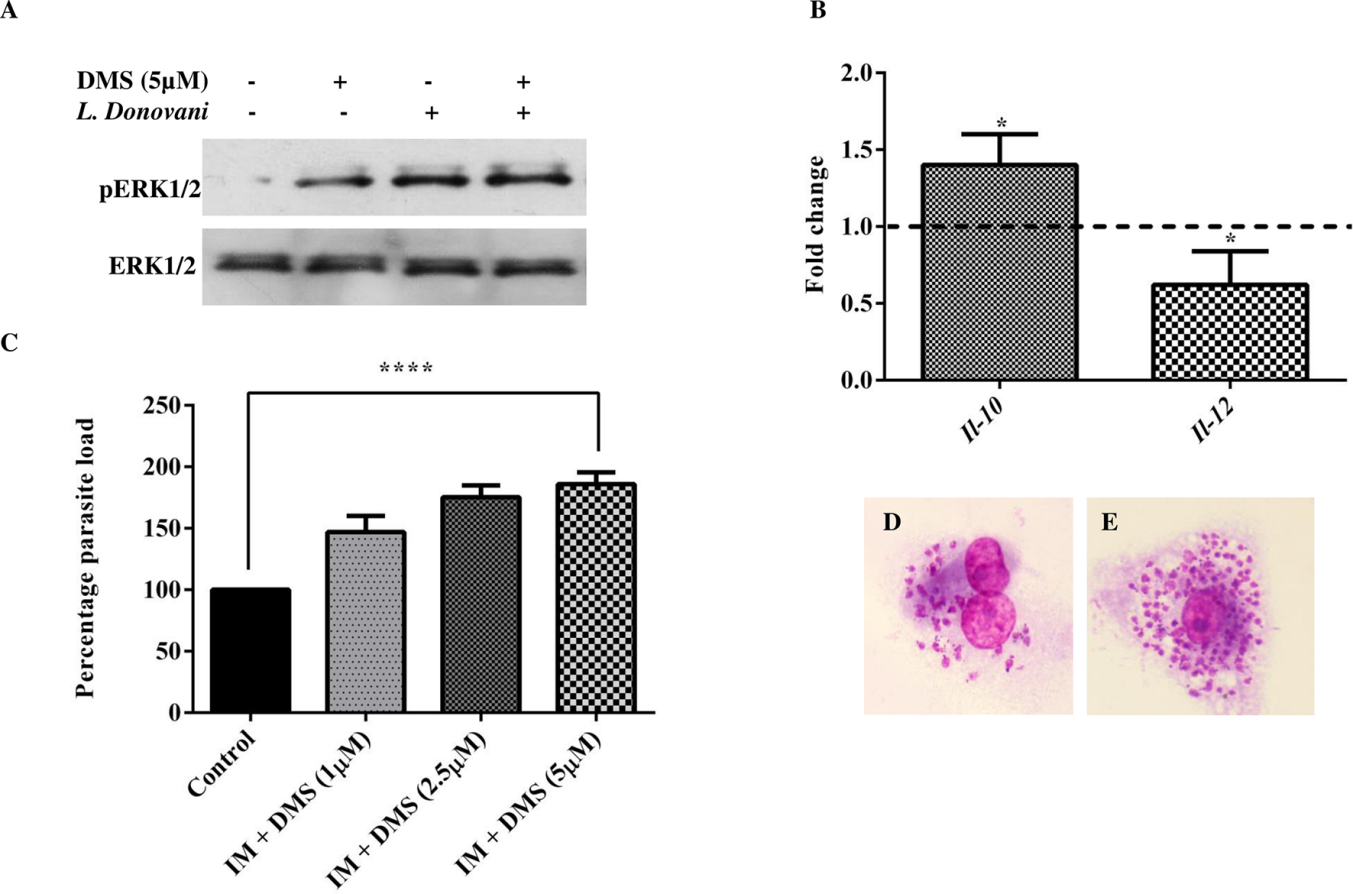
Pro-leishmanial effects of Sphingosine kinase inhibition. TDM were cultured in six-well plates in the presence or absence of *L*. *donovani* infection (MOI  =  1:10) for 6 h, TDM were washed to remove non-internalized parasites and incubated for next 42 h in presence and absence of Sphingosine kinase inhibitor, DMS. **A.** Western Blot showing phosphorylation of ERK1/2 and total ERK1/2. **B**. The graphic indicates the fold change of mRNA levels of IL-10 and IL-12 in DMS (5 μM) pre-treated infected macrophages in comparison to untreated infected macrophages by real-time PCR. Relative quantification was performed by the comparative Ct method (△△Ct). The data is a representation of mean ± SD from three independent experiments. Giemsa stained *L*. *donovani* infected TDM, **C**. The % parasite load was determined by direct counting with an optical microscope. **D**. Control and **E**. DMS (5 μM) treated infected TDM. The data is representation of mean ± SD from three independent experiments. *, p< 0.05; ****, p < 0.0001.

To further validate our results, we checked for ERK1/2 and p38 phosphorylation levels cytokine expression, and parasite load in presence of sphingosine kinase inhibitor, DMS. In case of hMDM, DMS pretreatment increases ERK1/2 phosphorylation, while reduces p38 phosphorylation in infected hMDM (Figs 4A and 4B and S4A and S4B). Additionally, we found that DMS pretreatment in infected macrophages resulted in a significant increase in *Il*-*10* expression, whereas decreased *Il-12* expression was observed as compared to infected hMDM (S4C Fig). In uninfected and infected TDM pretreated with 5 μM DMS, we found an increase in ERK1/2 phosphorylation (Figs 5A and S3A). Additionally, cytokines expression were checked by both semi-quantitative PCR and real-time PCR and it was found that *Il*-*10* expression was increased while *Il-12* expression was decreased in DMS pretreated infected TDM (Figs 5B and S3B). We further evaluate the parasite load in presence of increasing dose of DMS and we found that DMS pretreatment leads to increase in parasite load in a dose-dependent manner with significant increase in parasite burden at 5 μM (Fig 5C–5E).

### Anti-inflammatory response of S1PR2 and S1PR3 antagonist during *Leishmania* infection in human macrophages

**Fig 6:**
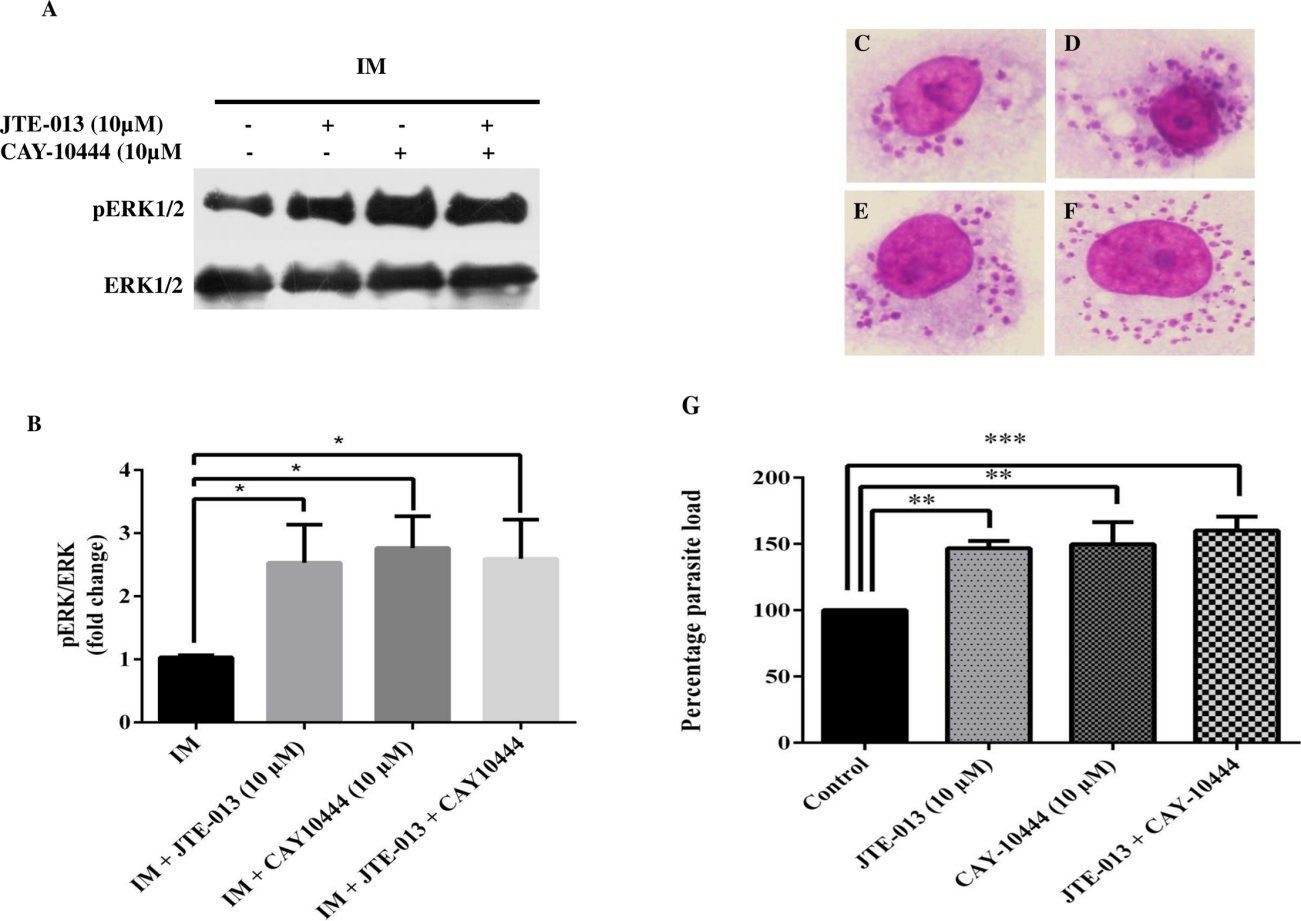
Anti-inflammatory effect on inhibition of S1PR2-3 in infected macrophages. TDM were cultured in six-well plates in the presence or absence of *L*. *donovani* infection (MOI  = 1: 10) for 6 h, TDM were washed to remove non-internalized parasites and incubated for next 42 h in presence and absence of JTE-013 and CAY-10444. **A.** Western Blot showing increase in the phosphorylation of ERK1/2 in presence of S1PR2 inhibitor (JTE-103) or S1PR3 inhibitor (CAY10444) or both in IM. **B.** Densitometric analysis of ERK1/2 in presence of JTE-013 and CAY10444 in infected and uninfected macrophages, after normalization with total ERK1/2. Giemsa stained *L*. *donovani* infected TDM. **C.** Control, **D.** JTE-013(10 μM) treated, **E.** CAY-10444 (10 μM) treated, **F.** JTE-013 (10 μM) and CAY10444 (10 μM) treated. **G.** The % parasite load was determined by direct counting with an optical microscope. The data is a representation of mean ± SD from three independent experiments. * p < 0.05, ** p < 0.01, ***, p < 0.001.

As shown in the previous experiment that the expression of S1PR2 and S1PR3 was decreased during *Leishmania* infection, we further checked for ERK1/2 phosphorylation and parasite load in presence of S1PR2 and S1PR3 specific inhibitors, JTE-013 and CAY10444, respectively [16,17]. We found that pretreatment with S1PR2 or S1PR3 inhibitors leads to a dose-dependent increase in the intracellular parasite load with a significant increase at higher dose i.e 10 μM (S5A and S5B Fig). In addition, ERK1/2 phosphorylation was also studied in JTE-013 (10 μM) and CAY10444 (10 μM) pretreated infected TDM alone and in combination. We found a significant increase in the phosphorylation of ERK1/2 in presence of these inhibitors, alone or in combination which further supports our study (Fig 6A and 6B). We further checked the parasite burden by pretreatment of both the inhibitors in combination or alone and we found that they both in combination contributed to further increase in the parasite burden (Fig 6C–6G).

**Fig 7:**
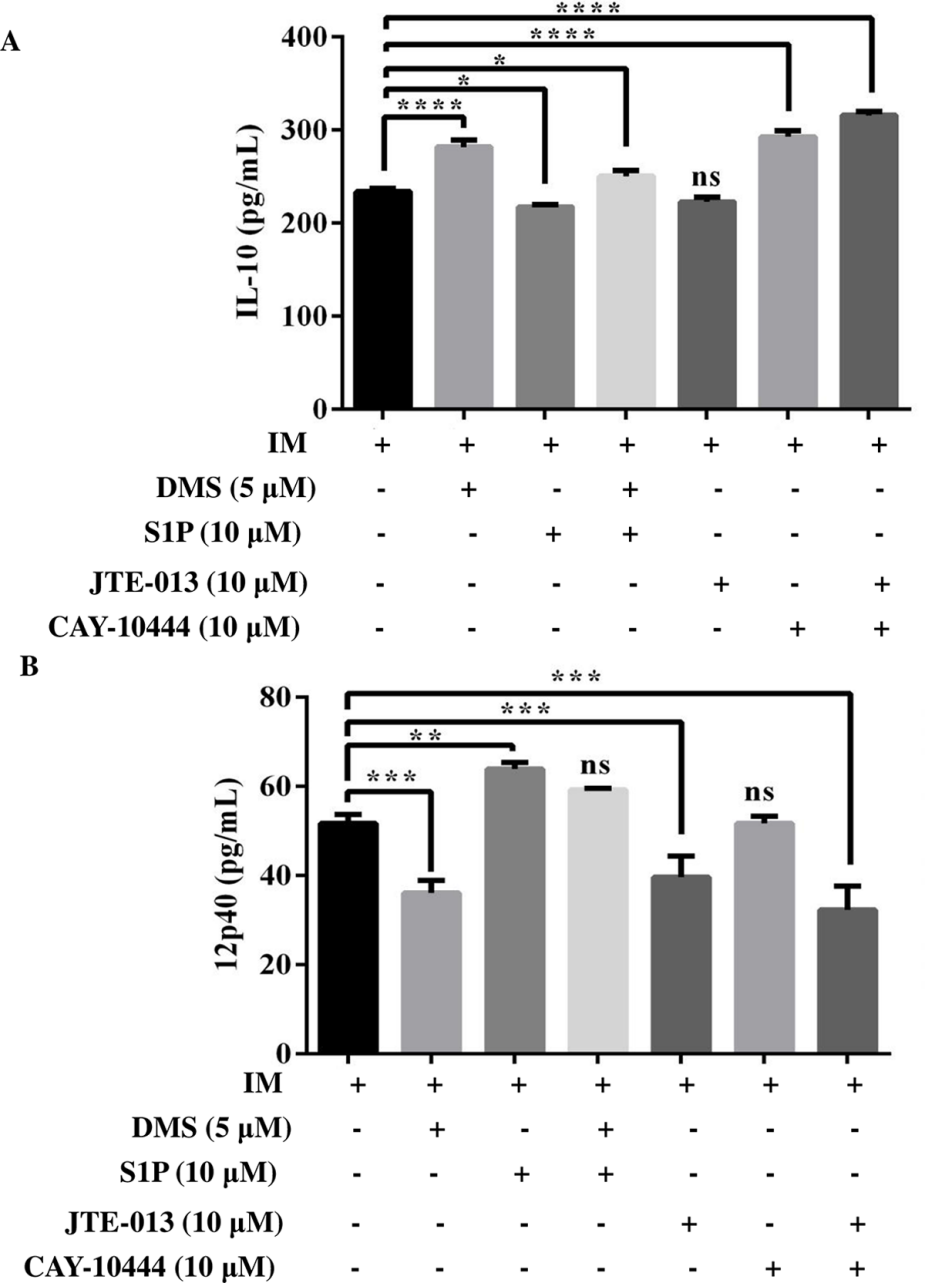
Cytokine production upon modulation of S1P signaling. TDM were cultured in the presence or absence of *L*. *donovani* infection (MOI  = 1: 10) for 6 h, TDM were washed to remove non-internalized parasites and incubated for next 42 h in presence of DMS, S1P, JTE-013, and CAY-10444. Cell-free supernatants were collected and **A**. IL-10, **B**. IL-12 secretion was measured by ELISA. The data is a representation of mean ± SD from three independent experiments. * p < 0.05, ** p < 0.01, ***, p < 0.001; ****, p < 0.0001; ns, non significant.

We next investigated secretion of IL-12 and IL-10 upon modulation of S1P signaling by DMS, S1P supplementation, and S1PR2-3 inhibition, alone or in combination. As expected, we found an increase in IL-12 secretion, whereas IL-10 secretion was decreased upon S1P supplementation (Fig 7A and 7B). In contrast, DMS pretreatment leads to increased IL-10 and decrease IL-12 (Fig 7A and 7B). In addition, we observed that by blocking S1PR3 there was a significant increase in IL-10 secretion, however, not significant changes in IL-10 in presence of S1PR2 inhibition was observed. However, on combinational doses of S1PR2-3 inhibitors, there was a more significant increase in IL-10 secretion (Fig 7A). Interestingly, by blocking S1PR2 we notice a significant decrease in the secretion of IL-12 in infected TDM while no significant changes were observed by blocking S1PR3. Furthermore, the secretion of IL-12 was further decreased in infected macrophages that were treated with S1PR2-3 inhibitors in combination (Fig 7B).

## Discussion

S1P signaling is emerging as a novel therapeutic target for numerous infectious diseases. Many studies have acknowledged the fact that S1P signaling plays a critical role in numerous infectious diseases and hence careful manipulation of the signaling might provide a breakthrough therapy. Hence in this study, we, for the first time, examined the role of S1P signaling in one of the most neglected tropical disease, Leishmaniasis.

SphK1 is a cytosolic enzyme that on activation translocated to the plasma membrane leading to S1P production [18]. Earlier studied showed that sphK plays a critical role in viral infection. Non-structural proteins from bovine viral diarrhea virus (BVDV) have been shown to binds and inactivate sphK1 activity in a time-dependent manner, which favors viral growth by inhibition of apoptosis in Madin–Darby bovine kidney (MDBK) cells [19]. SphK1 inhibition was also reported in dengue virus (DENV) infection in HEK-293 cells [20]. It was demonstrated that thymocytes from *Trypanosoma cruzi*-infected mice showed a decrease in the activity of sphK1 and sphK2 at mRNA level [21]. S1P has been shown to regulate the phosphorylation of several MAPK (mitogen-activated protein kinase) including ERK1/2 and p38. S1P induces the phosphorylation of both ERK1/2 and p38 with maximum activation between 10–30 min of stimulation [22,23]. S1P induced activation of p38 is shown to be mediated by S1PR2 in SVEC endothelial cells line and pre-incubation with JTE-013 leads to inhibit the ability of S1P to induced p38 phosphorylation [23].

ERK1/2 and p38 phosphorylation, are established biomarkers for the progression and suppression of *Leishmania* infection [4,24]. Increase in ERK1/2 phosphorylation induces IL-10 production that has been associated with disease progression [4]. Moreover, it was found that IL-10 neutralization restores p38 activation and promotes parasite clearance, in contrary, IL-12 neutralization increases parasite burden [25–27]. *Leishmania* infection inhibits p38 phosphorylation that reduces *Il-12* mRNA expression [28] and on the other hand, increase in p38 phosphorylation leads to IL-12 production that has been associated with disease suppression [4]. Altogether, IL-10 and IL-12 are considered to be important cytokines that regulate *Leishmania donovani* infection [29].

In this study, we observed that S1P results in decreased *Leishmania* induce ERK1/2 phosphorylation, while it increases p38 phosphorylation in macrophages. This further leads to induction of anti-leishmanial response by increase in IL-12 at mRNA and protein level, while a decrease in disease-promoting IL-10 at mRNA as well as in protein level, which altogether resulted in reduced parasite burden. DMS as a specific inhibitor of sphK which is reported by experiments done by other groups [30–32]. In our study, we also observed that DMS pretreatment inhibits sphK1 activity by inhibition of the phosphorylation of sphK1 in TDM (S6A Fig). Pharmacological inhibition of sphK by DMS or sphK1 specific siRNA has previously shown to decrease p38 phosphorylation in mouse macrophage cell line RAW264.7 [33]. Similarly, inhibition of sphK inhibition by same above approach in mice also has been shown to down-regulate pro-inflammatory cytokines [34]. These reports suggested regulation of MAPKs and cytokines by inhibition of sphK. We have shown that *Leishmania donovani* infection in human macrophages results in decreased sphK1 phosphorylation in a time-dependent manner. In addition, pharmacological inhibition of sphK1 by DMS have been shown to exacerbate the infection by increasing disease promoting ERK1/2 induced IL-10 secretion, while on the other hand, decreasing p38 activated IL-12 production.

Earlier, we showed that *Leishmania* infection significantly reduces the expression of *S1pr2-3* in infected macrophages. To further gain insight on the role of these receptors mediated signaling, we checked the expression of ERK1/2 in presence of S1PR2 and S1PR3 specific antagonist JTE-013 (10μM) and CAY10444 (10 μM). Earlier studies have shown that inhibition of S1PR2 by JTE-013 results in increased ERK1/2 phosphorylation [35,36]. In our study, we found that there was an increase in the expression ERK1/2 on inhibition of S1PR2-3, alone or in combination. We also confirm this by checking the intracellular parasite burden and we found that combinational does of both the inhibitors increase the intracellular parasite burden in a very significant manner. Furthermore, we also observed increased IL-10 secretion in infected macrophages upon S1PR3 inhibition and combination doses of S1PR2 and S1PR3 inhibitor, but no observable changes were seen upon S1PR2 inhibition. Interestingly, inhibition of S1PR2 significantly decreases IL-12 secretion but no significant decrease was found upon S1PR3 inhibition. Notably, combinational doses of both these inhibitors significantly decrease IL-12 secretion and which may contribute to the increased parasite survival as observed by increase in parasite load. Hence this study suggests that S1PR2-3 inhibition reciprocally regulates IL-10/IL-12 balance during *Leishmania* infection which altogether makes macrophages more susceptible to *Leishmania* infection.

**Fig 8:**
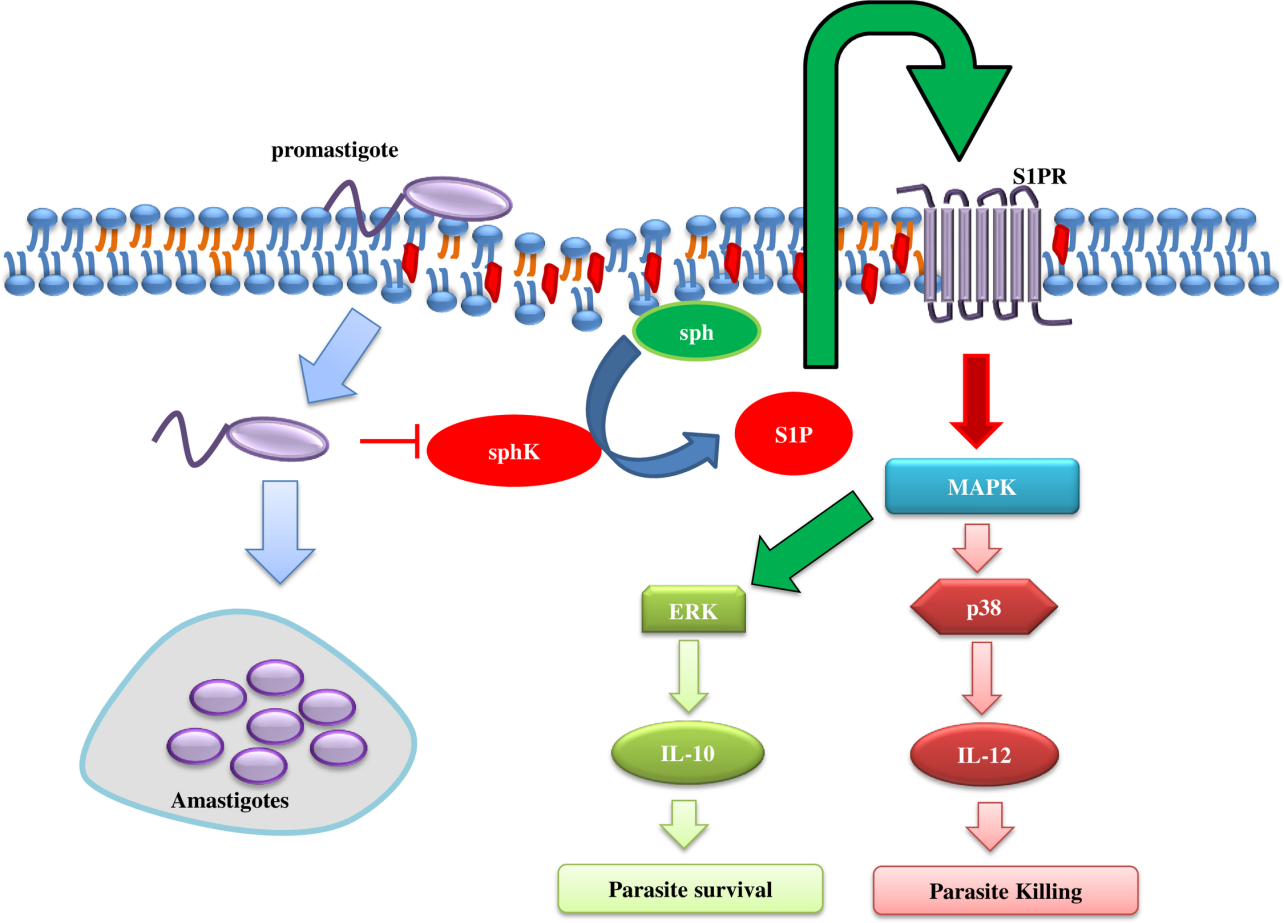
Schematic diagram of the anti-leishmanial response of S1P. S1P leads to the decrease in *L*. *donovani* induced ERK1/2 pathways and activation of p38 pathway that limits the infection. Infection of *L*. *donovani* leads to decrease in sphK1 phosphorylation leading to decrease in S1P that favors parasite growth.

In summary, we showed that S1P signaling plays a protective role in *Leishmania* infection and S1PR2-3 can be considered as novel and attractive therapeutic target against leishmaniasis. Our study suggested S1P mediated signaling abolishes *Leishmania* induce ERK1/2 phosphorylation resulting in low parasite load, on the other hand, S1P induces activation of p38 pathway that leads to IL-12 production for further clearance of the intracellular parasite (Fig 8). Additionally, blockage of S1PR2-3 mediated signaling by specific inhibitors in alone and in combinational doses resulted in activation of the ERK1/2 pathway leading to IL-10 production and increase parasite load. Hence, our study thus provides a novel and important aspect of S1P signaling during *Leishmania donovani* infection that may be helpful for the generation of a new line of anti-leishmanial drugs.

## Supporting information

S1 Table Table for primer sequences. (TIF)

S1 Fig Sphingosine kinase 1 phosphorylation in uninfected and infected hMDM. hMDM were cultured in six-well plates in the presence or absence of *L*. *donovani* infection (MOI  = 1: 10) for 6 h, hMDM were washed to remove non-internalized parasites and incubated for next 42 h **A.** Western Blot showing phosphorylation of sphK1 and total sphK1 in uninfected macrophages (UIM) and infected macrophages (IM) **B.** Fold change in the phosphorylation of sphK1 during *L*. *donovani* infection after normalization with total sphK1. The data is representation of mean ± SD from two independent experiments **, p < 0.01.
(TIF)

S2 Fig Anti-leishmanial response of S1P. TDM were cultured in six-well plates in the presence or absence of *L*. *donovani* infection (MOI  =  1:10) for 6 h, TDM were washed to remove non-internalized parasites and incubated for next 42 h in presence and absence of S1P. **A.** Densitometric analysis of phospho-ERK1/2 in presence and absence of S1P in UIM and IM, after normalization with total ERK1/2. **B.** Densitometric analysis of phospho-p38 in presence and absence of S1P in UIM and IM, after normalization with total p38. **C.** EtBr stained 1.5% agarose gel for profiling of IL-10 and IL-12 in S1P treated and untreated UIM and IM by semi-quantitative RT-PCR. The data is representation of mean ± SD from three independent experiments ** p < 0.01.
(TIF)

S3 Fig Pro-leishmanial response of DMS. TDM were cultured in six-well plates and pretreated with DMS for 30 min and then cultured in the presence and absence of *L*. *donovani* infection (MOI  =  1:10) for 6 h, TDM were washed to remove non-internalized parasites and incubated for next 42 h in presence and absence of DMS. **A.** Densitometric analysis of phospho-ERK1/2 in presence and absence of DMS in UIM and IM, after normalization with total ERK1/2. **B.** EtBr stained 1.5% agarose gel for profiling of IL-10 and IL-12 in DMS treated and untreated UIM and IM by semi-quantitative RT-PCR. The data is representation of mean ± SD from three independent experiments. *p < 0.05.
(TIF)

S4 Fig ERK1/2 phosphorylation and cytokine expression in infected macrophages in presence of DMS, S1P or both in hMDM. hMDM were cultured in six-well plates in the presence or absence of *L*. *donovani* infection (MOI  =  1:10) for 6 h, hMDM were washed to remove non-internalized parasites and incubated for next 42 h in presence and absence of S1P, DMS or both. Western Blot showing **A.** phospho**-**ERK1/2 and total ERK1/2, **B.** Densitometric analysis of phospho-ERK1/2 in presence of DMS, S1P, or both in IM, after normalization with total ERK1/2. The data is representation of mean ± SD from two independent experiments **C.** The graphic indicates the fold change of mRNA levels of IL-10 and IL-12 in S1P treated infected macrophages in comparison to untreated infected macrophages by real-time PCR. Relative quantification was performed by the comparative Ct method (△△Ct). The data is representation of mean ± SD from two independent experiments *p < 0.05., ** p < 0.01.
(TIF)

S5 Fig Parasite load upon modulation of S1P signaling. TDM were cultured in six-well plates in the presence or absence of *L*. *donovani* infection (MOI  =  1:10) for 6 h, TDM were washed to remove non-internalized parasites and incubated for next 42 h in presence and absence of increasing doses of S1PR2 inhibitor, JTE-013, or S1PR3 inhibitor, CAY10444, DMS (5 μM), S1P (10 μM) or both. **A.** Percentage parasite load in presence of increasing doses of JTE-013. **B.** Percentage parasite load in presence of increasing doses of CAY10444. **C** Percentage parasite load in presence of DMS (5 μM), S1P (10 μM) or both. The data is a representation of mean ± SD from three independent experiments. ***, p < 0.001; ****, p < 0.0001** p < 0.01.
(TIF)

S6 Fig Inhibition of sphK1 by DMS and cell cytotoxicity assay. **A**. Western blot showing phosphorylation of sphK1 and total sphK1 in uninfected macrophages (UIM) in presence and absence of DMS at given concentration. **B**. To check for cell cytotoxicity by inhibitors used in the study, MTT assay was performed. Briefly, 1 X 10^5^ cells TDM were cultured on 96 wells plate in presence and absence of inhibitors such as JTE-013 (10 M), CAY10444 (10) and DMS (5 M) for 42 h. After given time MTT (Sigma–Aldrich) was applied at in dark following 4 h incubation at 37°C. The MTT containing medium was replaced with 100 μl of isopropanol-HCl (0.1N) and kept at 37°C for 10 min to solubilize the formazan crystals. The samples were transferred to 96-well plates and the absorbance of the converted dye was measured at 570nm. The percent cell viability of the control (non treated) cells was taken as 100%. The data is a representation of mean ± SD from three independent experiments.
(TIF)
