# Supplementary figures and images for: Sphingosine-1-phosphate signaling in Leishmania donovani infection in macrophages
Source: PLoS Negl Trop Dis. 2018 Aug 17;12(8):e0006647. doi: 10.1371/journal.pntd.0006647 (PMC6118390; doi:10.1371/journal.pntd.0006647)

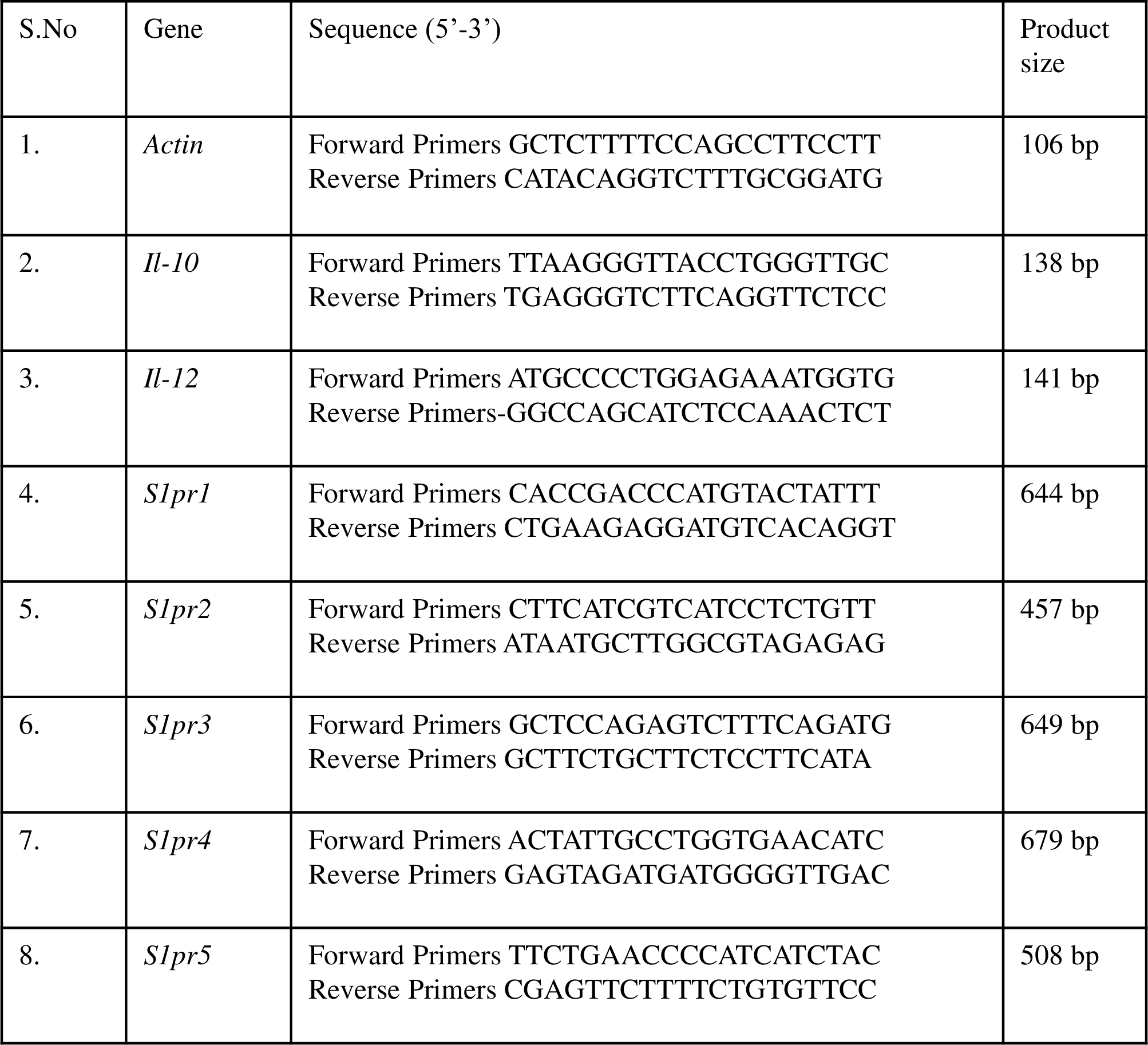

Supplement: S1 Table — (TIF) [file pntd.0006647.s001.tif]

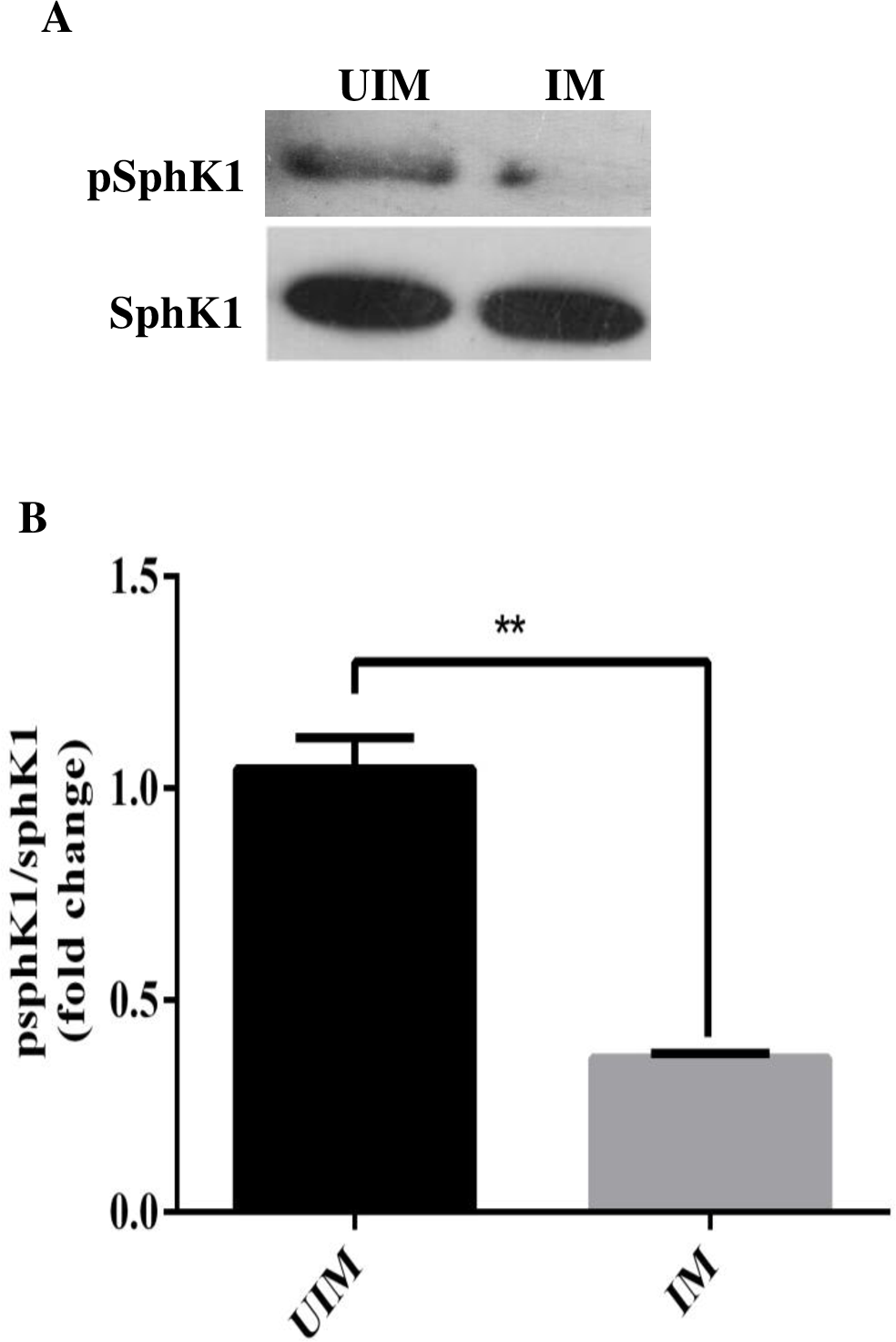

Supplement: S1 Fig — hMDM were cultured in six-well plates in the presence or absence of L. donovani infection (MOI = 1: 10) for 6 h, hMDM were washed to remove non-internalized parasites and incubated for next 42 h A. Western Blot showing phosphorylation of sphK1 and total sphK1 in uninfected macrophages (UIM) and infected macrophages (IM) B. Fold change in the phosphorylation of sphK1 during L. donovani infection after normalization with total sphK1. The data is representation of mean ± SD from two independent experiments **, p < 0.01. (TIF) [file pntd.0006647.s002.tif]

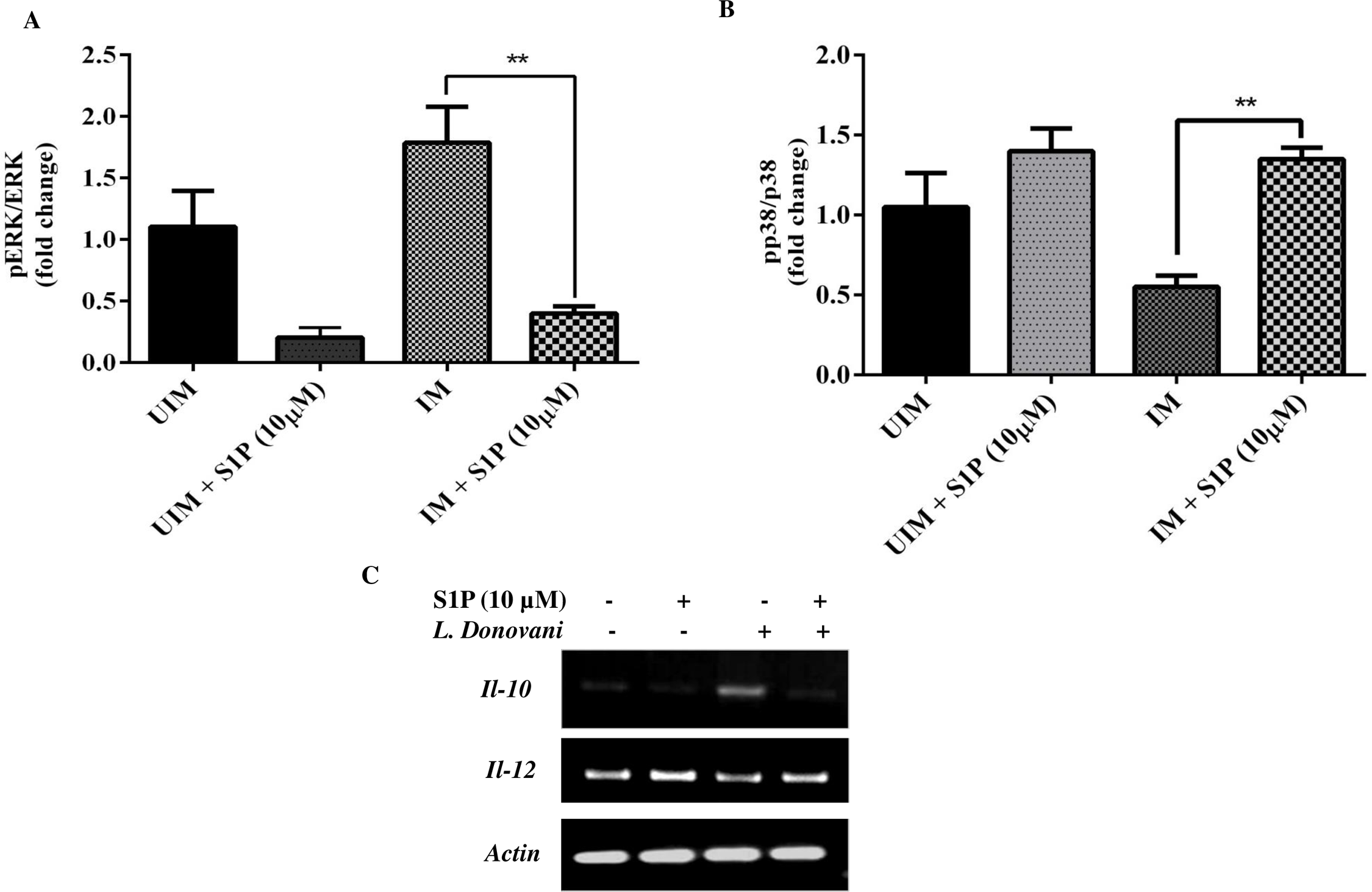

Supplement: S2 Fig — TDM were cultured in six-well plates in the presence or absence of L. donovani infection (MOI = 1:10) for 6 h, TDM were washed to remove non-internalized parasites and incubated for next 42 h in presence and absence of S1P. A. Densitometric analysis of phospho-ERK1/2 in presence and absence of S1P in UIM and IM, after normalization with total ERK1/2. B. Densitometric analysis of phospho-p38 in presence and absence of S1P in UIM and IM, after normalization with total p38. C. EtBr stained 1.5% agarose gel for profiling of IL-10 and IL-12 in S1P treated and untreated UIM and IM by semi-quantitative RT-PCR. The data is representation of mean ± SD from three independent experiments ** p < 0.01. (TIF) [file pntd.0006647.s003.tif]

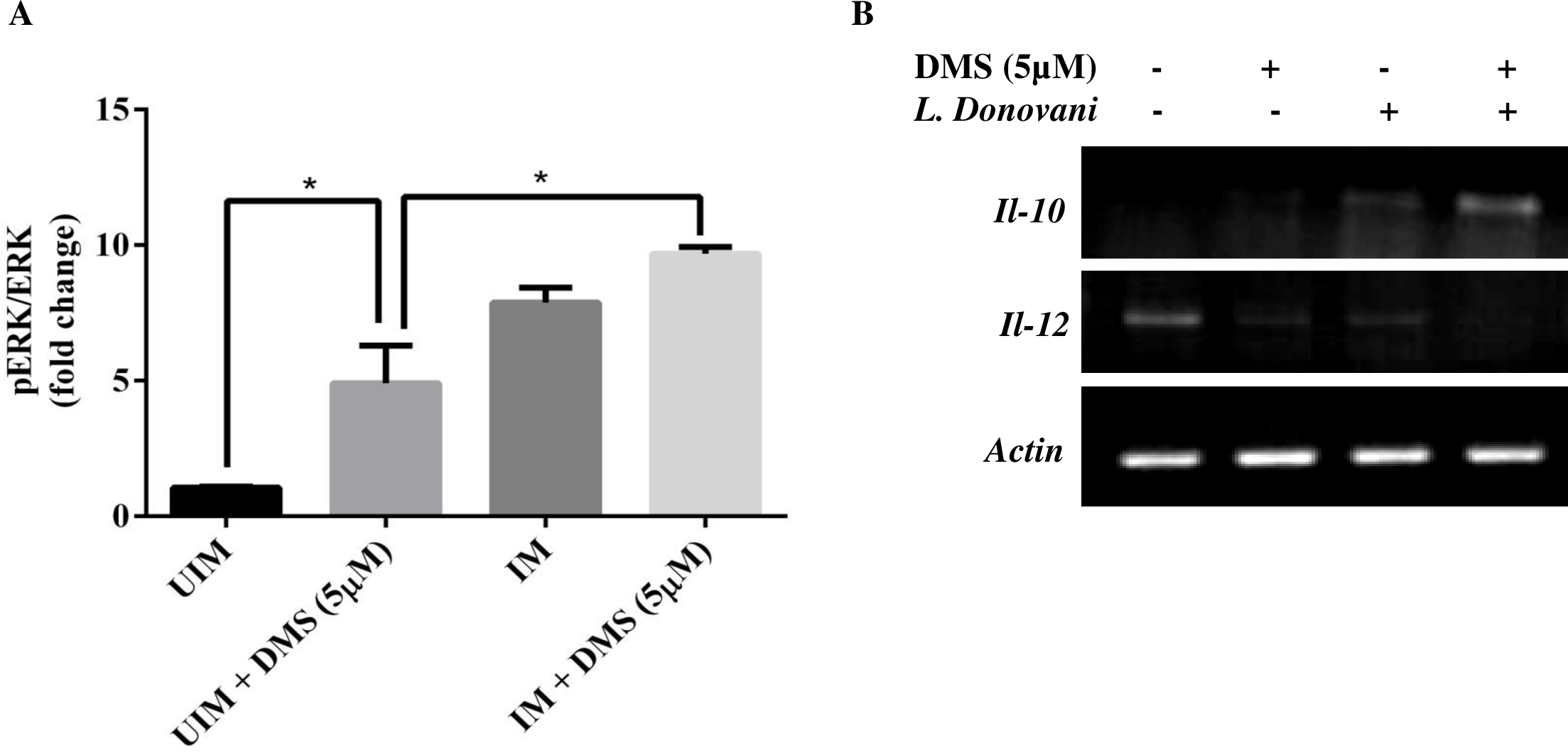

Supplement: S3 Fig — TDM were cultured in six-well plates and pretreated with DMS for 30 min and then cultured in the presence and absence of L. donovani infection (MOI = 1:10) for 6 h, TDM were washed to remove non-internalized parasites and incubated for next 42 h in presence and absence of DMS. A. Densitometric analysis of phospho-ERK1/2 in presence and absence of DMS in UIM and IM, after normalization with total ERK1/2. B. EtBr stained 1.5% agarose gel for profiling of IL-10 and IL-12 in DMS treated and untreated UIM and IM by semi-quantitative RT-PCR. The data is representation of mean ± SD from three independent experiments. *p < 0.05. (TIF) [file pntd.0006647.s004.tif]

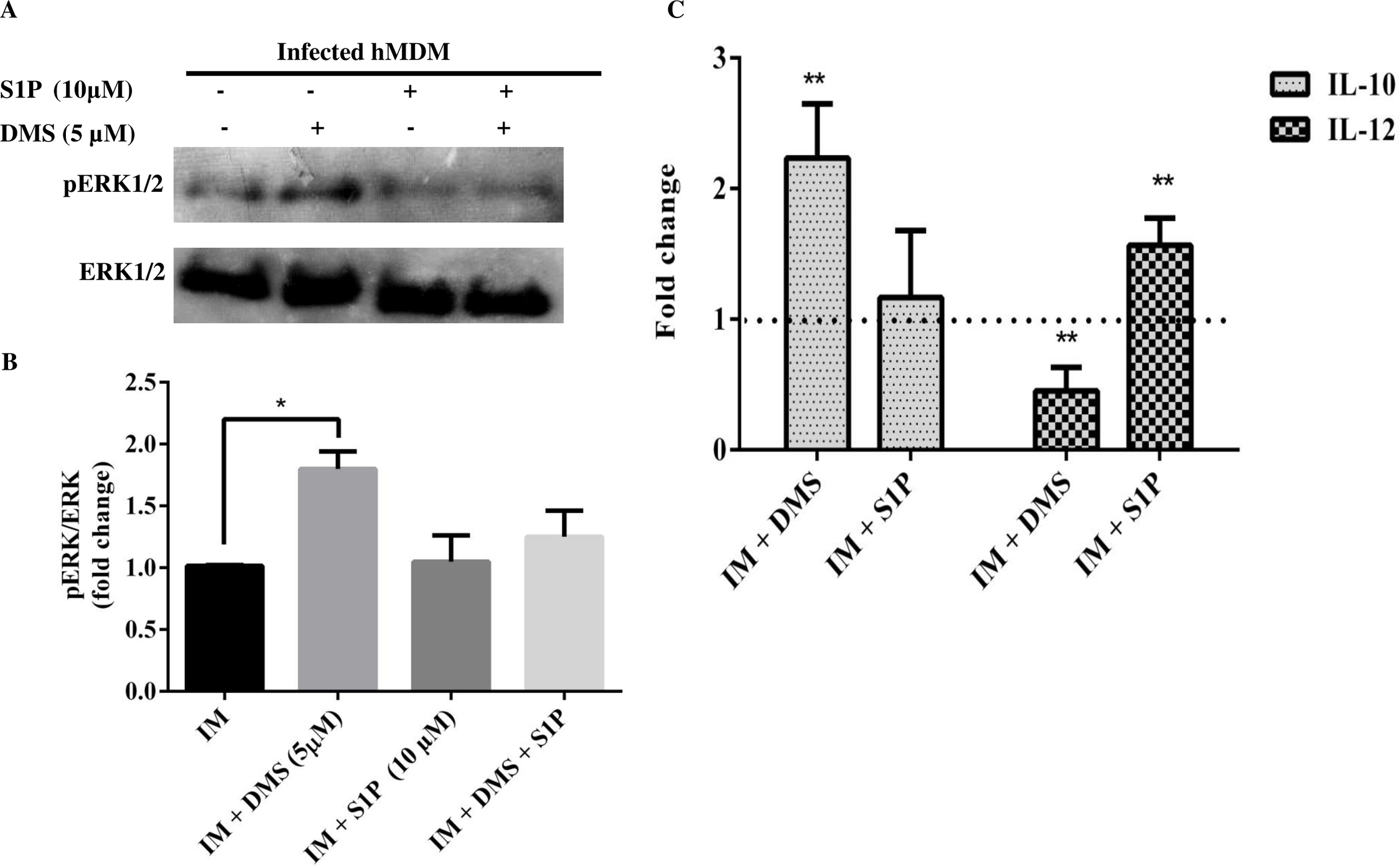

Supplement: S4 Fig — hMDM were cultured in six-well plates in the presence or absence of L. donovani infection (MOI = 1:10) for 6 h, hMDM were washed to remove non-internalized parasites and incubated for next 42 h in presence and absence of S1P, DMS or both. Western Blot showing A. phospho-ERK1/2 and total ERK1/2, B. Densitometric analysis of phospho-ERK1/2 in presence of DMS, S1P, or both in IM, after normalization with total ERK1/2. The data is representation of mean ± SD from two independent experiments C. The graphic indicates the fold change of mRNA levels of IL-10 and IL-12 in S1P treated infected macrophages in comparison to untreated infected macrophages by real-time PCR. Relative quantification was performed by the comparative Ct method (△△Ct). The data is representation of mean ± SD from two independent experiments *p < 0.05., ** p < 0.01. (TIF) [file pntd.0006647.s005.tif]

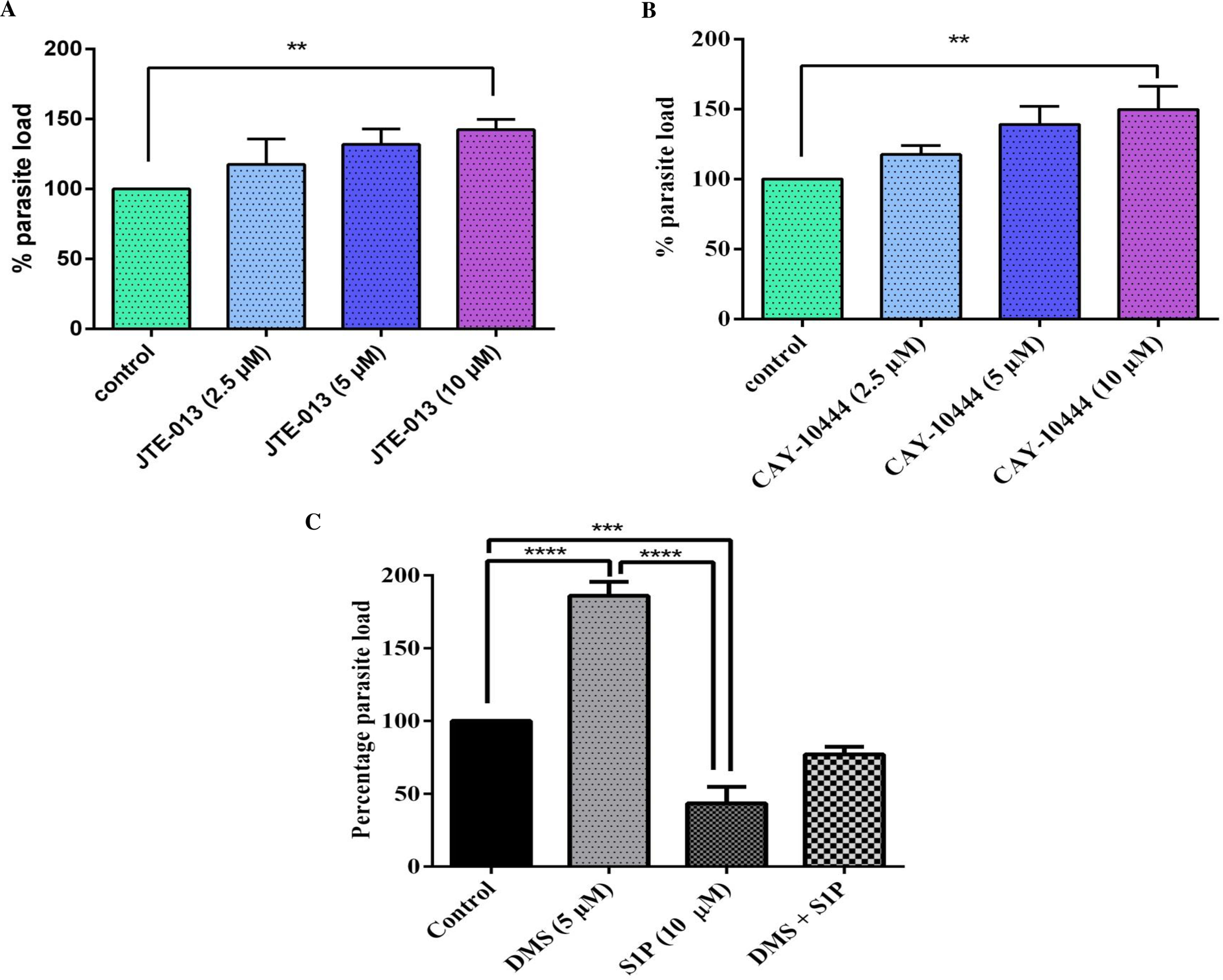

Supplement: S5 Fig — TDM were cultured in six-well plates in the presence or absence of L. donovani infection (MOI = 1:10) for 6 h, TDM were washed to remove non-internalized parasites and incubated for next 42 h in presence and absence of increasing doses of S1PR2 inhibitor, JTE-013, or S1PR3 inhibitor, CAY10444, DMS (5 μM), S1P (10 μM) or both. A. Percentage parasite load in presence of increasing doses of JTE-013. B. Percentage parasite load in presence of increasing doses of CAY10444. C Percentage parasite load in presence of DMS (5 μM), S1P (10 μM) or both. The data is a representation of mean ± SD from three independent experiments. ***, p < 0.001; ****, p < 0.0001** p < 0.01. (TIF) [file pntd.0006647.s006.tif]

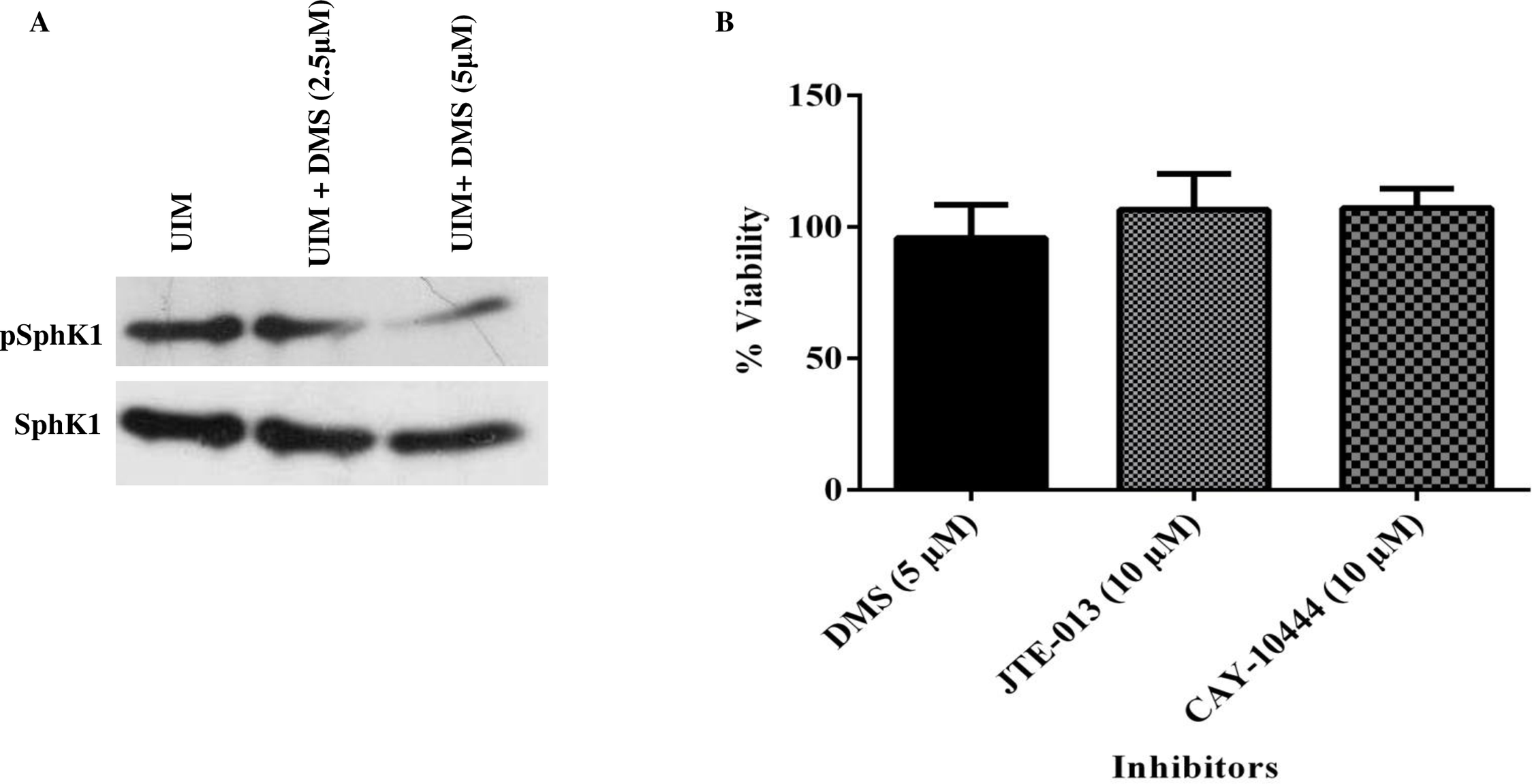

Supplement: S6 Fig — A. Western blot showing phosphorylation of sphK1 and total sphK1 in uninfected macrophages (UIM) in presence and absence of DMS at given concentration. B. To check for cell cytotoxicity by inhibitors used in the study, MTT assay was performed. Briefly, 1 X 105 cells TDM were cultured on 96 wells plate in presence and absence of inhibitors such as JTE-013 (10 M), CAY10444 (10) and DMS (5 M) for 42 h. After given time MTT (Sigma–Aldrich) was applied at in dark following 4 h incubation at 37°C. The MTT containing medium was replaced with 100 μl of isopropanol-HCl (0.1N) and kept at 37°C for 10 min to solubilize the formazan crystals. The samples were transferred to 96-well plates and the absorbance of the converted dye was measured at 570nm. The percent cell viability of the control (non treated) cells was taken as 100%. The data is a representation of mean ± SD from three independent experiments. (TIF) [file pntd.0006647.s007.tif]
